# Oxygen isotope effects during microbial sulfate reduction: applications to sediment cell abundances

**DOI:** 10.1038/s41396-020-0618-2

**Published:** 2020-03-09

**Authors:** E. Bertran, A. Waldeck, B. A. Wing, I. Halevy, W. D. Leavitt, A. S. Bradley, D. T. Johnston

**Affiliations:** 1 Department of Earth and Planetary Sciences, Harvard University, Cambridge, MA USA; 2 Department of Geological Sciences, University of Colorado Boulder, Boulder, CO USA; 3 Department of Earth and Planetary Sciences, Weizmann Institute of Science, Rehovot, Israel; 4 Department of Earth Sciences, Dartmouth College, Hanover, NH USA; 5 Department of Chemistry, Dartmouth College, Hanover, NH USA; 6 Department of Biological Science, Dartmouth College, Hanover, NH USA; 7 Department of Earth and Planetary Sciences, Washington University in St. Louis, St. Louis, MO USA; 8 Division of Biology and Biomedical Sciences, Washington University in St. Louis, St. Louis, MO USA

**Keywords:** Biogeochemistry, Biogeochemistry

## Abstract

The majority of anaerobic biogeochemical cycling occurs within marine sediments. To understand these processes, quantifying the distribution of active cells and gross metabolic activity is essential. We present an isotope model rooted in thermodynamics to draw quantitative links between cell-specific sulfate reduction rates and active sedimentary cell abundances. This model is calibrated using data from a series of continuous culture experiments with two strains of sulfate reducing bacteria (freshwater bacterium *Desulfovibrio vulgaris* strain Hildenborough, and marine bacterium *Desulfovibrio alaskensis* strain G-20) grown on lactate across a range of metabolic rates and ambient sulfate concentrations. We use a combination of experimental sulfate oxygen isotope data and nonlinear regression fitting tools to solve for unknown kinetic, step-specific oxygen isotope effects. This approach enables identification of key isotopic reactions within the metabolic pathway, and defines a new, calibrated framework for understanding oxygen isotope variability in sulfate. This approach is then combined with porewater sulfate/sulfide concentration data and diagenetic modeling to reproduce measured ^18^O/^16^O in porewater sulfate. From here, we infer cell-specific sulfate reduction rates and predict abundance of active cells of sulfate reducing bacteria, the result of which is consistent with direct biological measurements.

## Introduction

A significant fraction of biogeochemical cycling takes place within marine sediments and is driven by microbial activity [1, 2]. Microbial communities exhibit physiological [3, 4], biogeochemical, and phylogenetic [5, 6] complexity. Quantifying the distribution and growth rate of metabolically active cells is fundamental to understanding these environments [7, 8], particularly when considering cell-specific activity—the unit used to bridge natural observations with laboratory studies [9, 10].

**Fig. 1:**
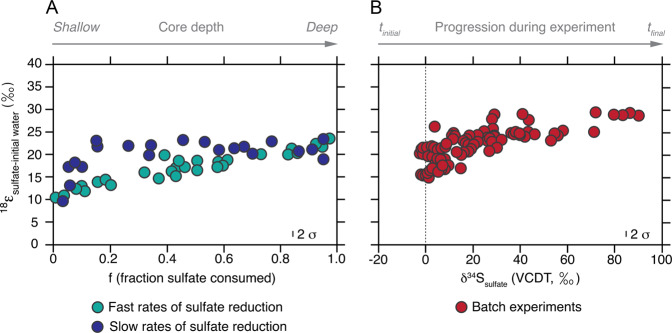
Compilation of MSR driven oxygen isotope effects. **a** Isotopic offset between porewater sulfates and ambient water compositions (compiled in [26], expressed in units of per mil, ‰) plotted as a function of fraction of sulfate consumed (f). Porewater with low relative sulfate reduction rates exhibiting strong non-linearity (dark blue circles), whereas settings capturing elevated sulfate reduction rates show a linear trend (green circles). **b** Isotopic offset between porewater sulfates and ambient water compositions and sulfur isotopic compositions (expressed relative to the international standard, VCDT, expressed in units of per mil, ‰) during recent microbial sulfate reduction batch experiments [17, 19, 24, 25, 28]).

In certain cases, cell-specific metabolic rates can be inferred via the inorganic chemical signatures of that activity. One such example, and central to this study, are the laboratory calibrated stable isotope effects associated with microbial sulfate reduction (MSR) [11, 12]. This approach is long-standing concerning the sulfate sulfur isotopic composition [11–15], but less explored in the case of sulfate oxygen isotope ratios [16–18]. Broadly, the oxygen isotopic composition of sulfate is interpreted to reflect sulfate consumption via MSR and sulfate regeneration through microbial sulfide oxidation and sulfur disproportionation [16, 18–22]. This is depicted in detail in Fig. 1. The figure shows the sulfate oxygen isotopic composition in porewaters (Panel a) and pure cultures (Panel b) undergoing active MSR, and highlights the role of sulfate reduction rates in the development of this oxygen isotopic signature. Recently, this interpretation has been extended to include the biochemistry of MSR and its specific role in modern marine environments, highlighting the potential of sulfate oxygen isotopic compositions to refine paleoenvironmental reconstructions [19, 21, 23–30]. Implicit in this new approach is isolating the reductive and oxidative fluxes within the MSR biochemistry.

We aim to quantitatively link the MSR biogeochemistry and its oxygen isotope effect. Mathematical frameworks have been developed to establish quantitative links between cell-specific parameters (i.e., rate [17, 19, 20, 24]) and the temperature and pH-sensitive [31] equilibrium oxygen isotope exchange between intracellular sulfur species and ambient water [29, 32–35]. This equilibrium oxygen isotope effect places a critical oxygen isotope equilibration step within the dissimilatory sulfate reduction pathway (Fig. S1) and serves as a marker of intracellular recycling—one that further carries important energetic consequences for adenosine triphosphate (ATP) budgets. In parallel to the challenges of constraining the role of isotopic equilibration between water and other cellular components (phosphate, sulfite, sulfate, etc.), there also exists the potential for a series of step-specific kinetic/equilibrium isotope fractionations associated with ion transport, complexation, and reduction/oxidation reactions. Kinetic oxygen isotope fractionations are often acknowledged [24], with isotope effects suggested to be small but pervasive [17] throughout the MSR network [24]. Together then with the additional complexity of sedimentary dynamics (advection, diffusion, and other metabolisms), the precise interpretation of sulfate oxygen isotopic compositions in porewaters and the water column is challenging.

In what follows we demonstrate that the oxygen isotopic composition of porewater sulfate tracks the abundance of active sulfate reducing bacteria in sediments. We first present results from a series of pure culture chemostat (*chem*ical environment in *stat*ic) experiments covering a range of growth rates and ambient sulfate concentrations using two strains of sulfate reducing Bacteria (the freshwater *Desulfovibrio vulgaris* strain Hildenborough and the marine *Desulfovibrio alaskensis* strain G-20). We use these data to calibrate an isotope model for MSR ^18^O/^16^O fractionation rooted in reaction thermodynamics. This model is adapted from a similar model focused on describing MSR sulfur isotope fractionation [12]. Our version establishes a quantitative link between the MSR-driven ^18^O signature in extracellular sulfate and cell-specific sulfate reduction rates (csSRR). Finally, with an understanding of the direct relationship between bulk sulfate reduction rates and csSRR, we show that cell abundances can be extracted from porewater sulfate ^18^O/^16^O profiles, and we use a well-studied marine sedimentary environment for which full diagenetic model analyses are available as an illustrative example. What results is an isotopic tool that can be used alongside molecular techniques in modern environments.

## Methods

*Desulfovibrio vulgaris* (strain Hildenborough) and *Desulfovibrio alaskensis* (strain G-20) were grown in stirred continuous culture vessels (i.e., chemostats) at 24 °C. The growth vessel was continuously purged with high-purity, oxygen-free N_2_:CO_2_ (90:10) gas, maintaining anaerobic conditions. The gas flow swept biogenic sulfide out of the reactor into a series of zinc acetate traps. The pH in the culture vessel was kept constant (7.00 ± 0.02) via a pH-probe activated titration pump. For the experiments with *D. vulgaris*—run over a large range of growth rates—sulfate was present in excess (28 mM sulfate in the influent media). For the chemostat experiments with G-20, the reactor sulfate concentration was set to 0.5, 1, 2, and 5 mM for different experiments. Sulfate was captured as barite (BaSO_4_) and preserved for isotope analyses. In all experiments, lactate was the limiting substrate and added in stoichiometric proportions to facilitate 50% sulfate consumption (see [11, 36] for expanded experimental details). Additional experimental data, expanded discussion of the methods, sulfur isotopic measurement methods, sulfur isotopic data, and sulfur fractionation factors, as well as general considerations are published elsewhere [11, 36]. Note that during these experiments, sulfate samples were taken only when the chemostats reached steady state conditions (i.e., when mass and sulfur isotope influx and outflux balance out). Details on how steady state was determined for each chemostat experiment are available in the original publications for these experiments [11, 36].

Barite precipitates were filtered and washed with deionized water, oven-dried at 50 °C overnight, and stored at room temperature in sealed containers post sampling and prior to isotopic analysis. Precipitate rinses using weak acid removed any co-existing, oxygen-bearing phases such as barium carbonate. For the analysis, ~0.4 mg of barite was weighed into silver boats with excess ground glassy carbon. Samples were run in duplicates where possible. Isotopic compositions were measured via combustion on a TC/EA, where barite transfers oxygen to CO, connected to a Finnigan Delta V configured in continuous flow mode. Each analytical run (~30 unknowns) included compositionally distinct standards (NBS-127, IAEA-SO5, and IAEA-SO6, plus two internal lab standards) with an average reproducibility of ±0.2‰ in *δ*^18^O (where the δ notation used here corresponds to the ‰ difference in the sulfate oxygen isotopic ratio and the Vienna Standard Mean Ocean Water: *δ*^18^O = (^18^R_sample_/^18^R_VSMOW_ – 1) × 1000) based on standard reproducibility. The oxygen isotopic composition of ambient water during these experiments was measured via laser spectrometer (Picarro L2140i) at the University of Washington.

## Results

**Fig. 2:**
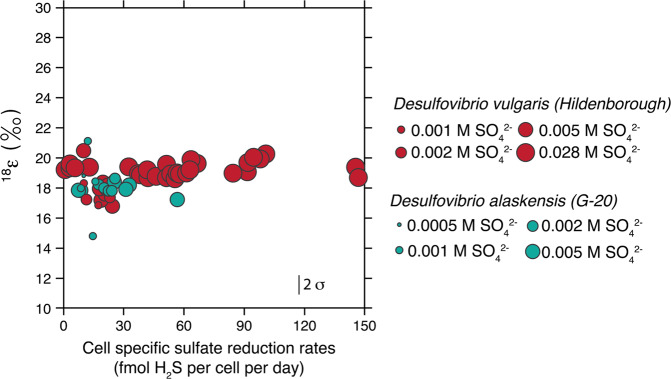
Oxygen isotope composition of extracellular sulfate during *Desulfovibrio vulgaris* (red circles) and *Desulfovibrio alaskensis* (blue circles) chemostat experiments. The size of the circles corresponds to the sulfate concentrations in the feed media, as shown in the figure’s legend. Error is shown as a 2*σ* in the lower right corner of the figure and applies to all data points. Also shown is the sulfate-water oxygen isotopic equilibrium fractionation (gray line).

Measured metabolic rates ranged from 0.9 to 146.7 fmol H_2_S per cell per day when sulfate concentrations were held at 28 mM. For the low sulfate experiments (0.5–5 mM), rate was held constant at 15 (±5.3) and 22 (±8.9) fmol H_2_S per cell per day for *D. vulgaris* and *D. alaskensis*, respectively. Net sulfur isotope fractionation (expressed as ^34^ε: the isotopic offset between sulfate and sulfide) ranged from 17.2 to 56.5‰ for the high sulfate, variable rate experiments. For the low sulfate experiments, ^34^ε varied from 0.1 to 11.4‰ for *D. alaskensis* and from 24.2 to 27.5‰ for *D. vulgaris* (Fig. 2 and S2). In all experiments, aqueous sulfide concentrations in the reactor were below detection when using colorimetric assays ([H_2_S] < 1 μM).

Water normalized oxygen isotopic composition of extracellular sulfate for all chemostat experiments are in Table S1 and presented in Fig. 2. Values for the isotopic offset between the oxygen isotopic composition of pools of interest, epsilon (^18^ε; when no subscript shown, the offset is between the oxygen isotopic composition of water and sulfate) range from 14.8 to 21.1‰ relative to experimental water. Combined data yielded an average value of 18.6‰ (1 *σ* = 1.1‰, *n* = 57) with greater variability at lower metabolic rates, as shown in Fig. S3. Also cast in Fig. S3 is a Gaussian curve overlying the data to highlight the nature of the distribution.

## Discussion

There exists a rich literature of sulfate oxygen isotope data from environmental settings ([26] and references within), and pure culture experiments ([19, 24, 25, 28, 17]), and a framework for how MSR drives sulfate oxygen isotopic signatures is emerging. However, much is still lacking with respect to our knowledge of intracellular conditions, and the isotopic consequences therein. Further, knowledge about the thermodynamic and energetic state of a cell that gives rise to a characteristic oxygen isotope effect in sulfate is fully absent. It is the goal of this study to assess the contribution of physiological and environmental controls to net sulfate oxygen isotope compositions, while incorporating the extent to which the water oxygen isotopic composition anchors this biosignature. These details will enable the translation of a sulfate oxygen isotope measurement into predictions for active cell counts in modern environmental settings.

We first interpret experimental data in the context of MSR. As presented in Fig. S3, there is no significant rate-dependence of ^18^ε (well-approximated by a Gaussian distribution). It follows that variability in tested parameters (csSRR, ambient sulfate concentrations, and/or strain of bacterial sulfate reducer, at least among the two tested here (Table S2)), carries no statistically significant relationship to oxygen isotope composition of sulfate (*p* ≥ 0.9). This suggests that neither environmental nor physiological controls dominate the observed oxygen isotope effect—a more sophisticated approach is therefore necessary.

## The biochemical network of MSR

Net isotope effects are composites of multiple, intermediate isotope effects. The main metabolic reactions that influence the MSR-driven sulfate oxygen isotopic composition are at first presumed to be the same reactions for concomitant sulfur isotope system (Fig. S1) [12, 37, 38]. Sulfate is imported into the cell via transporters. Intracellular sulfate is activated by ATP sulfurylase, which adds an ATP to sulfate, and forms adenosine phosphosulfate (APS). This APS is then reduced to sulfite (SO_3_^2−^) by APS reductase. In a classic MSR reaction network, the terminal step is the reduction of sulfite to sulfide (H_2_S) [39], which is transported out of the cell and/or allowed to equilibrate with the extracellular environment (Eq. 1)

1 $${\mathrm{SO}}_{4\,{\mathrm{out}}}^{2 - } \rightleftharpoons {\mathrm{SO}}_{4\,\,{\mathrm{in}}}^{2 - } \rightleftharpoons {\mathrm{APS}} \rightleftharpoons {\mathrm{SO}}_3^{2 - } \rightleftharpoons {\mathrm{H}}_2{\mathrm{S}}.$$

Here, “out” and “in” correspond to extracellular and intracellular environments, respectively. The two isotope systems (sulfur and oxygen) share core biochemistries, yet, a modified reaction network is required to better represent the oxygen isotope system. Various equilibria with water play key roles in the oxygen isotope composition of intracellular species: the near instantaneous sulfite-water isotopic equilibrium is perhaps the most crucial [29, 34]. Any potential oxygen isotope exchange between more reduced sulfur intermediates downstream from sulfite (S_2_O_3_^2−^, S_3_O_6_^2−^, etc., as has been reported before, giving rise to the so-termed “trithionate pathway” [40]), or sulfide reoxidation reactions would effectively be erased at the sulfite stage. Thus, these effects are not evident in the final oxygen isotopic composition of extracellular sulfate. It is also for that reason that our reaction network of choice (Eq. 1) does not include any sulfur redox intermediate, and we adhere to the simpler network presented. Rather than sulfide serving as the terminal product of the MSR pathway, as is the case for sulfur isotopes, the oxygen isotope network terminates with sulfite at isotopic equilibrium with water. The latter effectively acts as an infinite isotopic reservoir and leads to the following simple oxygen isotope network

2 $${\mathrm{SO}}_{4\,\,{\mathrm{out}}}^{2 - } \rightleftharpoons {\mathrm{SO}}_{4\,\,{\mathrm{in}}}^{2 - } \rightleftharpoons {\mathrm{APS}} \rightleftharpoons {\mathrm{SO}}_3^{2 - } \rightleftharpoons {\mathrm{H}}_2{\mathrm{O}}.$$

This relies on the fundamental presumption that the residence time of sulfite in the cell is long enough to allow isotopic equilibration with water. As modeled (details below), residence time estimates are on the order of 10^−5^ s (Fig. S4), whereas the measured sulfite-water oxygen isotope equilibrium is only known to be faster than ‘seconds’ [29, 34]. Future experiments can and should target the determination of more precise isotopic exchange rates for both sulfite and phosphate with water.

Recent experimental work demonstrates no oxygen isotope exchange between APS and water [41, 42]. During APS formation, the enzyme-driven reaction invests an ATP to produce APS and is thought to preserve the ^18^O composition of substrate sulfate. The reaction proceeds through the nucleophilic attack of the sulfate oxygen on the α-phosphate of ATP, producing APS and pyrophosphate [43]. In the reverse direction (APS to sulfate), sulfate is enzymatically liberated following the nucleophilic attack of the pyrophosphate oxygen on the sulfur atom of APS [44]. This recovers the ATP invested in the forward reaction. The isotopic composition of the APS pool is then a mass balance on the flux from sulfate activation (catalyzed by ATP sulfurylase) and from sulfite oxidation (catalyzed by APS oxidoreductase). The oxygen isotope composition of APS in the oxidative direction is a product of the sulfite-water isotopic equilibrium (three out of four oxygens in APS) and the phosphate group (one out of four oxygens in APS) [29, 34].

The regeneration of sulfate from the decomposition of APS can also happen abiotically as a hydrolysis reaction in weak acid [44]. This generates sulfate but differs from the enzymatic reaction in oxygen isotope effect, reaction rate, and cellular energy consequence. Two reactions are possible: unimolecular elimination or bimolecular displacement. Each bears specific sulfate oxygen isotopic consequences, owing to the specific bond broken (either the sulfur-oxygen or the phosphorus-oxygen bond). Both reactions occur simultaneously but contribute unequally to the total abiotic APS hydrolysis [44]. Bimolecular displacement proceeds via the nucleophilic attack of a water oxygen on the phosphorus in APS that breaks a phosphorus-oxygen bond. The ^18^O/^16^O of the resulting sulfate derives entirely from APS. This reaction contributes to only 10% of the total abiotic APS hydrolysis, as shown by early experiments [44]. Unimolecular elimination of sulfur trioxide results in the breaking of a sulfur-oxygen bond and produces sulfate with an oxygen isotopic composition issued from both water and phosphate—three from the sulfate within the APS complex and one with a water-buffered phosphate composition. Compared with the bimolecular displacement scenario, the isotopic composition of APS is then diluted due to the contribution of the water oxygen isotopic composition (see Eq. S11 for details). This reaction is responsible for 90% of total APS hydrolysis [44]. The potential for abiotic and enzymatic reactions happening in parallel with variable isotopic selectivity is included in our modeling approach.

## Model approach

The net isotope effect is then captured by a balance of kinetic and equilibrium isotope fractionation factors weighed by the degree of reversibility of a given reaction (i.e., the ratio of backward to forward fluxes [12]). During the steady state transformation of an oxygen-bearing reactant (r) to an oxygen-bearing product (p), the net isotope effect $({\,\!}^{{\mathrm{18}}}{\upalpha}_{{\mathrm{r}},{\mathrm{p}}}^{{\mathrm{net}}})$ is expressed as:

3 $${\,\!}^{{\mathrm{18}}}{\upalpha}_{{\mathrm{r}},{\mathrm{p}}}^{{\mathrm{net}}} \,=\, \left( {{\,\!}^{18}{\upalpha}_{{\mathrm{r}},{\mathrm{p}}}^{{\mathrm{eq}}} \,-\, {\,\!}^{18}{\upalpha}_{{\mathrm{r}},{\mathrm{p}}}^{{\mathrm{kin}}}} \right)\times{\mathrm{f}}_{{\mathrm{p}},{\mathrm{r}}} \,+\, {\,\!}^{18}{\upalpha}_{{\mathrm{r}},{\mathrm{p}}}^{{\mathrm{kin}}},$$

where ${\,\!}^{{\mathrm{18}}}{\upalpha}_{{\mathrm{r}},{\mathrm{p}}}^{{\mathrm{eq}}}$ is the reaction equilibrium isotope fractionation factor, and ${\,\!}^{{\mathrm{18}}}{\upalpha}_{{\mathrm{r}},{\mathrm{p}}}^{{\mathrm{kin}}}$ is the kinetic isotope fractionation factor of the reaction in the forward direction (from r to p). Note that ^18^*α*^eq^ is the ratio of backward to forward ^18^*α*^kin^ values. Reaction reversibility is expressed as f_p,r_ and is directly linked to the thermodynamics of the reaction itself [12]; a full derivation of *f*, and its linkage to free energy is in Eqs. S1–S4. Importantly, this model uses specific predicted electron carriers (FAD, for example) and redox pairs for each reaction within the dissimilatory network (see ref. [45] for a sensitivity test). The specific experimental substrate (i.e., lactate in the current study) controls (together with the other substrates and products) the energetics of the overall reaction and, therefore, the cell-specific sulfate reduction rate. However, it is the energetics of the intracellular reactions, which are insensitive to the identity of the specific organic substrate, that determine the reversibility of reactions and the net isotopic fractionation associated with them.

Interpreting the relationship between *f* values and isotopic fractionation is straightforward. When *f*_p,r_ is close to unity, the backward and forward fluxes balance out and equilibrium conditions dominate: ${\,\!}^{{\mathrm{18}}}{\upalpha}_{{\mathrm{r}},{\mathrm{p}}}^{{\mathrm{net}}}$ = ${\,\!}^{{\mathrm{18}}}{\upalpha}_{{\mathrm{r}},{\mathrm{p}}}^{{\mathrm{eq}}}$. Equilibrium isotope estimates with ambient water are available for three key species present in our model (sulfate, sulfite, and phosphate) as a function of temperature and in one case, pH (sulfite) [29, 31, 34]. These values (see Eqs. S5–S7), then serve as the ^18^α^eq^ for those components within the model. When *f*_p,r_ approaches zero, the reaction is dominantly unidirectional in the forward direction, and kinetic isotope effects dominate: ${\,\!}^{{\mathrm{18}}}{\upalpha}_{{\mathrm{r}},{\mathrm{p}}}^{{\mathrm{net}}}$ = ${\,\!}^{{\mathrm{18}}}{\upalpha}_{{\mathrm{r}},{\mathrm{p}}}^{{\mathrm{kin}}}$. Much of the uncertainty in solving for the source of a net isotopic fractionation within the MSR oxygen isotope system is the limited understanding of the kinetic oxygen isotope effects associated with each reaction. Calibrating these isotope fractionation factors is one of the goals of this study.

For a linear reaction network at steady state like MSR, the net isotopic fractionation at any upstream step is a nested expression incorporating the isotope fractionations of downstream reactions [12, 22]. Net oxygen isotope fractionations between the primary reactant (sulfate) and terminal product (water) encompasses the isotopic effects of all intermediate steps in the reaction sequence (Eqs. S8–S12). Each of these intermediate reaction steps have multiple possible mechanisms for the same bulk chemistry and incorporate multiple *f* and *α* values (kinetic and equilibrium). The proposed model for sulfate oxygen isotopic compositions during MSR carries additional input constraints. As the sulfur and oxygen atoms share much of the MSR dissimilatory pathway (compare Eqs. 1 and 2), published sulfur isotope solutions [12] from these same experiments [11, 36] must be consistent with any proposed model solution for oxygen isotopes in sulfate. This requires that step-specific *f* values from the sulfur isotope solution [12] be the same as for the oxygen model where the reactions overlap, satisfying the mass balance requirement. This is accommodated within our model results.

## Calibrating the thermodynamic isotope model

It is challenging to uniquely solve for the oxygen isotope effect associated with MSR given the number of unknown variables. As the system is under-determined, we solve for the magnitude of each undescribed isotope effect with a nonlinear least-squares regression for each individual chemostat experiment (*n* = 57). This yields unique values for each isotope effect that, when plugged back into our thermodynamic metabolic model, minimize the sum of squared residuals while also satisfying requirements for the sulfur isotope model solution (*f* values shared with sulfur isotope solutions of same experimental data point).

One additional layer of complexity requires consideration. As described above, the isotopic composition of intracellular sulfate includes contributions from both the enzyme-catalyzed decomposition of APS (to sulfate) as well as abiotic APS hydrolysis (to sulfate). The proportion of these fluxes relative to one another is captured as *F*_APS_. When *F*_APS_ is equal to 1, the reaction is dominated by the enzyme ATP sulfurylase. Conversely, when *F*_APS_ is equal to 0, the step is dominated by abiotic APS hydrolysis (assuming the 90:10 isotopic split between competing abiotic pathways noted above). The simplest first approximation is that enzyme catalysis outpaces the abiological reaction rate, that is, *F*_APS_ equals 1. However, a sensitivity test of this presumption comes in a parallel minimization routine where the value of *F*_APS_ is variable. We consider both solutions.

**Fig. 3:**
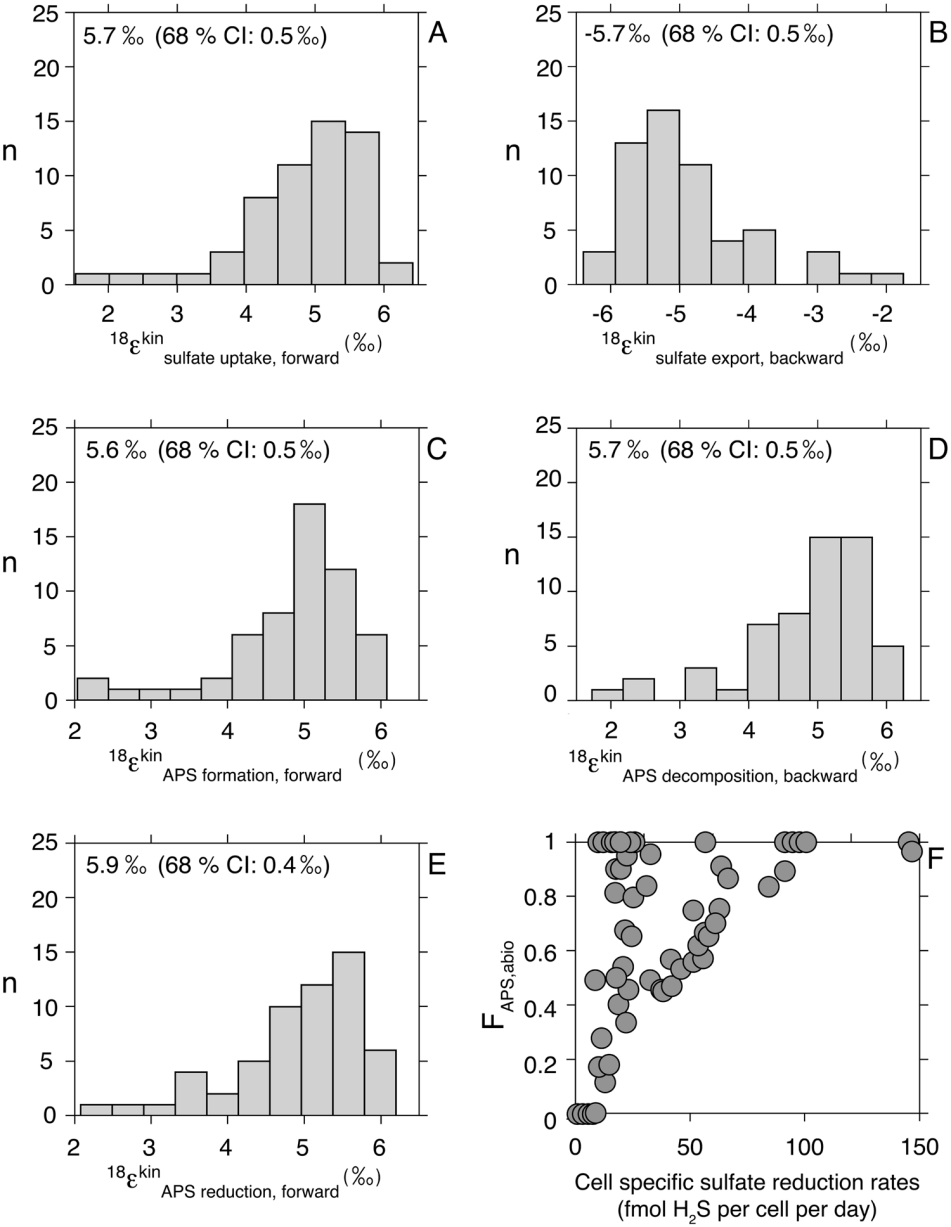
Distribution of step-specific oxygen kinetic isotope effects (in order in **a**–**e**: sulfate uptake, sulfate export, APS formation, enzymatic APS decomposition, and APS reduction), as well as contribution of APS hydrolysis (**f**) to intracellular sulfate, resulting from the least-square analysis for each experiment (shown in Fig. 2). Note that, for simplicity, kinetic effects are shown in epsilon space (with units of ‰), as opposed to unitless alpha values. Also shown are median values of the distributions, as well as 68% confidence intervals, owing to the asymmetry in the distribution of the data.

An initial output of our model is a set of predicted oxygen kinetic isotope fractionation factors when APS is only cycled enzymatically (*F*_APS_ = 1). Some of these predictions can be directly compared with published estimates (Table 1, Fig. 3). For example, the best-fit estimate for the oxygen kinetic isotope effect during APS reduction to sulfite (${\,\!}^{{\mathrm{18}}}{\upvarepsilon}_{{\mathrm{APS}}\;{\mathrm{red}}.,\;{\mathrm{forward}}}^{{\mathrm{kin}}}$) is within error of previous experimental work [17]. Interestingly, the oxygen kinetic isotope fractionation factors during sulfate uptake in the forward and reverse directions (${\,\!}^{{\mathrm{18}}}{\upvarepsilon}_{{\mathrm{sulfate}}\;{\mathrm{uptake}}}^{{\mathrm{kin}}}$, and $^{{\mathrm{18}}}{\upvarepsilon}_{{\mathrm{sulfate}}\;{\mathrm{export}}}^{{\mathrm{kin}}}$) bear opposite signs and indistinguishable magnitudes. Thus, the corresponding oxygen equilibrium isotope effect is 0‰ so that when f for sulfate uptake approaches unity this step effectively bears no net isotope effect. Finally, the oxygen isotope fractionation factors associated with sulfate activation to APS (both in the forward and reverse directions, ${\,\!}^{{\mathrm{18}}}{\upvarepsilon}_{{\mathrm{APS}}\;{\mathrm{formation}}}^{{\mathrm{kin}}}$ and ${\,\!}^{{\mathrm{18}}}{\upvarepsilon}_{{\mathrm{enzymatic}}\;{\mathrm{APS}}\;{\mathrm{decomposition}}}^{{\mathrm{kin}}}$) are also of the same magnitude and sign. This enzymatically-driven reaction involves the formation and breaking of a phosphorus-oxygen bond linking the sulfate and phosphate groups in APS. It involves small changes in the oxidation state of the central oxygen, consistent with the fact that our analysis yielded nonzero oxygen isotope fractionation estimates. These are mathematical predictions, however, and deserve systematic analysis via well-calibrated experiments.

**Table 1:** List of step-specific kinetic oxygen isotope effects for which the current study solves, expressed as *α* notation. The reaction step, description, and identification in the analysis are shown.

Reaction Step	Identification in the analysis	Description
Sulfate transport	^kin^α_sulfate uptake_	Sulfate uptake (forward direction)
	^kin^α_sulfate export_	Sulfate release (backward direction)
Sulfate activation to APS	^kin^α_APS activation, forward_	APS sulfurylase (forward direction)
	^kin^α_enzymatic APS decompositon, backward_	APS sulfurylase (backward direction)
APS reduction to sulfite	^kin^α_APS reduction, forward_	APS reductase (forward direction)

**Fig. 4:**
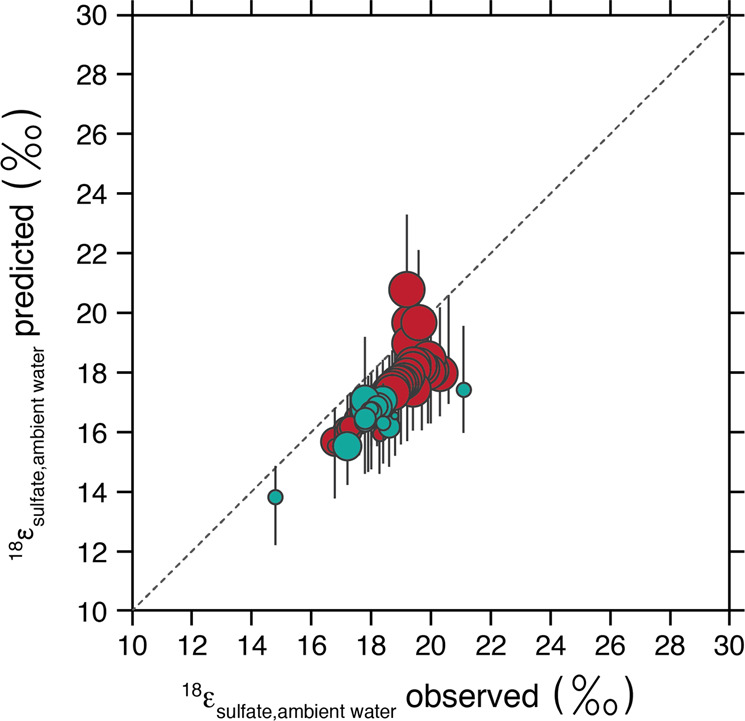
Oxygen isotope fractionation factor ^18^ε_sulfate-initial water_ predicted by our metabolic model compared with values measured during the chemostat experiments. Error bars correspond to the 68% confidence interval on kinetic oxygen isotope effects and F_APS_ inferred via non-linear least-squares, centered around the median value of said effect. The legend used for the experimental data is the same as for Fig. 2.

The initial treatment presumed no abiological decomposition of APS to intracellular sulfate. As abiological reactions are not often considered in metabolic models of MSR, we approach the potential contribution of this reaction in a conservative fashion, evaluating results both with and without such a reaction. With the addition of a new fitting parameter (F_APS_), the goodness of the mathematical fits— measured again by the residual between prediction and measured—improves. Indeed, the increasing contribution of abiotic APS hydrolysis relative to enzymatic activity induces a change in *δ*^18^O_sulfate_ of up to ~5‰ (Fig. S5). At higher metabolic rates, best-fit FAPS values fall close to 1, implying full enzymatic control of the APS back-reaction to the intracellular sulfate pool (Fig. 3f, Figure S6 for confidence intervals). As csSRR decrease, best-fit F_APS_ values decrease towards full abiotic control on sulfate production from APS. This general trend makes intuitive sense if at low csSRR the residence time of intracellular APS increases and if hydrolysis is a first-order reaction on APS concentration. However, analysis of APS residence times is not in keeping with this intuition (Fig. S6). While we expected a characteristic non-linear decrease of APS residence times with increasing sulfate reduction rates, APS residence time appear to first increase with rate. The residence time reaches an apex of 8 × 10^–7^ s at csSRR of 40 fmol H_2_S per cell per day, then decreases non-linearly with increasing sulfate reduction rates. The only tangible conclusion is that the abiological control on APS decomposition depends on more than simply the concentration of APS. Specific mechanism(s) aside, using the estimated oxygen isotope fractionation factors and experiment-specific F_APS_ values, the model satisfies 93% of the experimental observations (using a 68% confidence interval on each oxygen kinetic isotope effect; Fig. 4). We use this calibration moving forward but note that it can and will be improved with additional data.

The possibility of APS hydrolysis carries one additional consequence for the bacterial cell that warrants discussion. As explained earlier, during sulfate activation to APS, one ATP is invested. In the backward direction, the final product of both enzymatic and abiotic reactions is sulfate, but ATP is only regenerated during enzymatic catalysis, leading to a minimized net loss of invested ATP when the reactions are under biological control. Conversely, during APS hydrolysis, ATP is not obviously regenerated. The contribution from hydrolysis— determined from our mathematical fit— generally increases as rate decreases, meaning the cell is becoming less energy efficient (Fig. 3f). That said, the minimum ATP budget required for a viable sulfate reducing bacterium (here, viability is defined in its most minimalist sense of maintenance without growth) is 4 × 10^−24^ moles of ATP per cell per second [46, 47]. This equates to a csSRR of 1.5 × 10^−4^ fmol H_2_S per cell per day, which is orders of magnitude smaller than those covered in the chemostat experiments. In this case, even the possible inefficiency is not obviously catastrophic.

## Inferring active sediment cell abundances (Aarhus Bay, DK)

With our experimentally calibrated model, we set out to explore the physiological and environmental controls on the *δ*^18^O in sulfate observed in modern marine porewaters. Recall that the model leans on isotopic information, as well as local thermodynamic setting (sulfate and sulfide) and csSRR. We first examine the general porewater setting in typical sedimentary environments, and then show how csSRR, and ultimately active cell abundances can be predicted from geochemical and isotopic information in a specific site in Aarhus Bay.

**Fig. 5:**
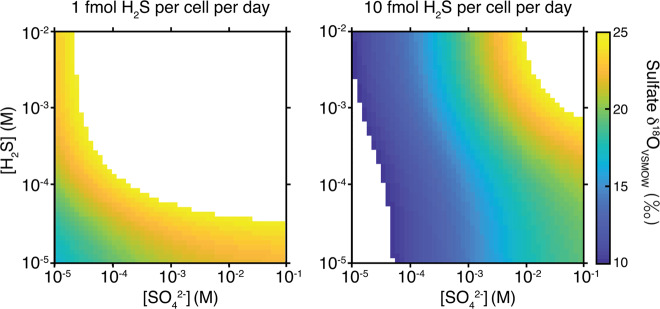
Field of values (focused on the range between +10 and +25‰) of *δ*^18^O_sulfate; extracellular_ for a range of extracellular sulfate and sulfide concentrations. On the left results are shown for cell-specific sulfate reduction rates at 1 fmol H_2_S per cell per day, on the right, results are shown for 10 fmol H_2_S per cell per day. For this analysis, we used median values of step-specific kinetic oxygen isotope effects inferred using non-linear least-squares analysis. Results expanded to reflect the 68 and 95% confidence intervals are shown in Fig. S7 in the Supplementary Material. Temperature, and the oxygen isotopic composition of ambient water and intracellular phosphate are adjusted to reflect porewater conditions. The back-reaction of APS to intracellular sulfate is set to be dominated by abiotic APS hydrolysis, as per our inferences from its relation to low cell-specific sulfate reduction rates (Fig. 2).

We predict *δ*^18^O values for marine porewater sulfate across relevant sulfate/sulfide concentration space as a function of typical environmental csSRR. This is captured in Fig. 5 at 1 and 10 fmol H_2_S per cell per day (see Fig. S7 for confidence intervals). The typical marine porewater sulfate *δ*^18^O range is between 10 and 25‰, so we focus on this range. We also tailored temperature-dependent isotopic equilibria with water (ambient water and intracellular phosphate oxygen isotopic compositions) to reflect general porewater conditions, and values of F_APS_ are taken from the rate-dependent fitting analysis.

At seafloor temperatures, there is a clear, nonlinear response in the *δ*^18^O of sulfate to changes in ambient sulfate and sulfide concentrations (Fig. 5). Higher concentrations of extracellular sulfur species are required for elevated sulfate *δ*^18^O values. Indeed, at high concentrations, reaction reversibility generally increases as a result of changes to the free energy (Eqs. S1–S4). When following a contour of constant *δ*^18^O, sulfate concentrations decrease, and sulfide concentrations increase. This covariance resembles trends deeper in sediment profiles, where the *δ*^18^O becomes invariant with changing sulfate/sulfide concentrations [26]. The sulfate *δ*^18^O prediction is also inversely correlated to csSRR—similar to that observed for the sulfur isotope system [11]. This is interpreted as the effect of growth rate on reaction reversibility (*f* values). Similar to the sulfur isotope effects at low growth rates and under steady state conditions, net forward and backward fluxes approach unity and the net oxygen isotope effect recorded in sulfate approaches the sulfate–water oxygen isotope equilibrium of ~25‰.

**Fig. 6:**
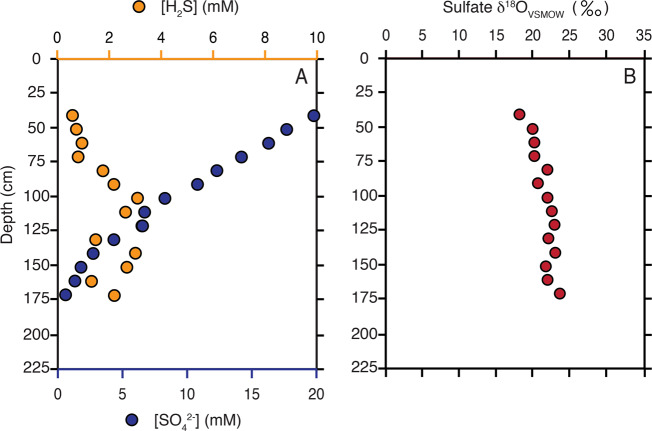
Measured [48] geochemical profiles in site M1 in Aarhus Bay. **a** Aqueous sulfate (dark blue circles) and sulfide (yellow circles) concentration depth profiles. Note the difference magnitude between the two profiles. **b** Depth-specific sulfate oxygen isotopic compositions.

**Fig. 7:**
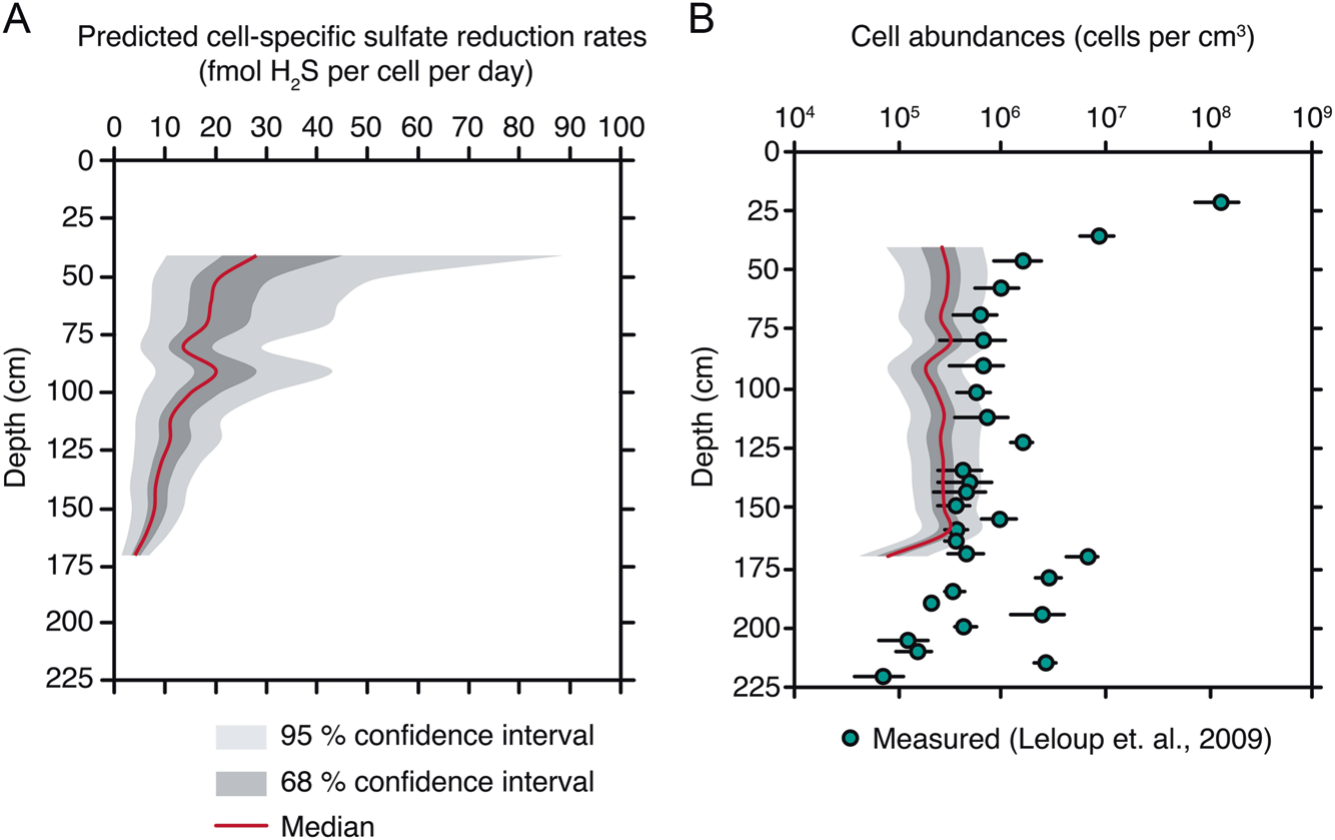
Predictions on depth- specific cell- specific sulfate reduction rates and active sulfate reducing bacteria cellular abundances. **a** Inferred depth-specific sulfate reduction rates in fmol H_2_S per cell per day, the red line corresponds to predictions using the median value of step-specific oxygen isotope effects, and the dark and light gray areas represent the corresponding 68 and 95% confidence interval, respectively. **b** Measured values for active sulfate reducing bacteria cellular abundances [49], and corresponding error, are shown as blue circles. Corresponding cellular abundance trends inferred by our model for oxygen isotope composition in sulfate are also shown. The red line corresponds to predictions using the median value for cell- specific sulfate reduction rates, and the gray areas correspond to 68 and 95% confidence intervals.

The model output can be directly related to an environmental porewater profile through an inversion of variables. Our cellular scale model solves for net oxygen isotope effects produced for given physiological (csSRR) and environmental (sulfate and sulfide concentrations) conditions. The inverse exercise is also possible: using environmental observations (sulfate and sulfide concentrations and observed sulfate oxygen isotopic compositions) to solve for physiology (csSRR). We target a sediment profile from the well-studied site (M1) in Aarhus Bay (Fig. 6) [48]. Environmental information (sulfate and sulfide concentrations, Fig. 6a) is used to infer csSRR required to reproduce measured *δ*^18^O in porewater sulfate. Specifically, for any given cell-specific metabolic rate, a corresponding *δ*^18^O sulfate value will form a contour that transects sulfate/sulfide concentration space (Fig. 5). From here, a minimization routine is run to find a best-fit between that oxygen isotope contour (as a function of rate) and the measured depth-specific sulfate and sulfide concentrations (Fig. 6a; fit in Fig. S8). This analysis results in a best-fit prediction for the depth- and cell-specific sulfate reduction rates (Fig. 7a).

Calculating active cell abundances then simply requires csSRR to be merged with diagenetic model rate estimates. This is done via a straightforward unit conversion (fmol H_2_S per cell per day) and incorporating the organoclastic sulfate reduction rate (μmol S per cm^3^ per year) from diagenetic model studies at the same site and depth [48]. This results in an estimate of active sedimentary cell abundances (cells per cm^3^). Aarhus Bay was specifically chosen for this comparison given that active sedimentary cell abundances were independently calculated using molecular tools [49]. Our results statistically overlap with those molecular estimates (Fig. 7b).

## Conclusions

Quantifying the distribution of actively metabolizing cells, their activity, and growth rates is fundamental to understanding the vigor of biogeochemical cycling [7, 8]. This work suggests that the MSR oxygen isotopic signature can be used to infer the abundance of active cells in marine sediments. We built an experimentally calibrated steady state isotope model rooted in reaction thermodynamics and kinetics that establishes quantitative links between csSRR, environmental conditions, and stable oxygen isotope effects.

Our calibration used data from chemostat experiments and a previously built steady state sulfur isotope model adapted to the MSR oxygen isotope system [12]. We explored the step-specific oxygen isotope effects required to explain observed sulfate oxygen isotopic compositions and employed a revised metabolic network reaction that included critical ties to water. This model also left open the possibility of abiological APS hydrolysis. We argue that the MSR-driven oxygen isotope effect measured in both pure cultures and porewater settings is predictable, and a function of physiological state (csSRR) and environmental conditions (sulfate and sulfide concentrations). Interestingly, at low metabolic rates, abiotic APS hydrolysis is in competition with enzymatic reactions. The chemical and isotopic consequences of this reaction are under-constrained and merit further testing. All together, however, it informs a physiologically based understanding of the isotopic, environmental, and thermodynamic controls on MSR-driven sulfate oxygen isotope fractionations in a manner that is fully consistent with sulfur isotope effects and holds between laboratory and sedimentary settings. Further, our model allows for in-depth insight into sulfite re-oxidation to sulfate. Specifically, we can determine the fraction of sulfate that is reset with respect to oxygen isotopes, and whose signature is effectively recorded in the final extracellular sulfate oxygen isotopic compositions. Further details on the computation of this value are described in the Supplementary Material. This fraction decreases with rates of cell-specific sulfate reduction, and with relative abundance of extracellular sulfate and sulfide concentrations (Fig. S9). When applied to global sulfate reduction rate estimates, the fraction of sulfate recycled will enhance our understanding of the environmental information enclosed in porewater sulfate *δ*^18^O trends in both modern and paleo-environments.

We tested the model against porewater data from a classic marine sediment site (site M1, Aarhus Bay). Our model effectively reproduces observed sulfate *δ*^18^O trends and allowed calculation of depth-dependent csSRR, as well as abundance of active cells of sulfate reducing bacteria. It should be noted, though, that sediments are dynamic environments, and that the information we are extracting corresponds only to the contribution of MSR to the sulfate oxygen isotope profile. Other processes, such as diffusion, advection, and other metabolic pathways, are not included but may play a central role. To that end, it is interesting to note that our full oxygen isotope analysis makes similar predictions for the ^34^S/^32^S of porewater sulfate (Fig. S10). Here, there is a systematic offset between model prediction and sedimentary data, which we interpret as a function of those physical processes noted above and the differing residence times of S and O in sulfate within a porewater setting. What remains to be tested is the potential role of other metabolic processes and/or highly variable physics (advection and diffusion); both of which are accessible through further experimental work and diagenetic modeling.

## Supplementary information

Supplementary Material

Supplementary Figure 1

Supplementary Figure 2

Supplementary Figure 3

Supplementary Figure 4

Supplementary Figure 5

Supplementary Figure 6

Supplementary Figure 7

Supplementary Figure 8

Supplementary Figure 9

Supplementary Figure 10

Supplementary Table 1

Supplementary Table 2
