# Supplementary material for: Oxygen isotope effects during microbial sulfate reduction: applications to sediment cell abundances
Source: ISME J. 2020 Mar 9;14(6):1508–19. doi: 10.1038/s41396-020-0618-2 (PMC7242377; doi:10.1038/s41396-020-0618-2)
Supplement: Supplementary file 1 — Supplementary Material [file 41396_2020_618_MOESM1_ESM.docx]

Supplementary Material for:

**Oxygen isotope effects during microbial sulfate reduction:**

**applications to sediment cell abundances**

E. Bertran^1^*, A. Waldeck^1^, B. A. Wing^2^, I. Halevy^3^, W. D. Leavitt^4^, A. S. Bradley^5^, D. T. Johnston^1^*

^1^Department of Earth and Planetary Sciences, Harvard University

^2^Department of Geological Sciences, University of Colorado Boulder

^3^Department of Environmental Sciences and Energy Research, Weizmann Institute of Science ^4^Department of Earth Sciences, Dartmouth College

^5^Department of Earth and Planetary Sciences, Washington University in St. Louis

*To whom correspondence may be addressed: emmabertran@fas.harvard.edu, johnston@eps.harvard.edu

**1 Supplementary information**

**The Wing and Halevy modified metabolic model**

The Wing and Halevy model (2014) is a quantitative descriptor for sulfur isotope fractionation during dissimilatory sulfate reduction. It explicitly links fractionation, reaction reversibility, and intracellular metabolite concentration. Reaction reversibility is itself linked to the thermodynamic driving force of the chemical transformation it characterizes, which ultimately establishes a direct link to environmental (extracellular sulfate, and sulfide concentrations), and physiological parameters (csSRR) to net isotope fractionation. Reaction reversibility is captured as:

$f_{p,r}= e^{\frac{\Delta G_{r}}{\mathrm{RT}}}$ , (S1)

where R is the gas constant, T is temperature (in Kelvin), and ΔG_r_ is the free energy change associated with the reactant/product reaction. The ΔG_r_ is defined as:

$\Delta G_{r}= \Delta G_{r}^{o}+RT \times ln\left( \frac{\Pi_{i}\left[ p_{i} \right]^{m_{i}}}{\Pi_{j}\left[ r_{j} \right]^{n_{j}}} \right)$ . (S2)

The ΔG_r_^o^ is the free energy change of the reaction at standard-state conditions, and m_i_ and n_j_ are the stoichiometric coefficients of the i^th^ product and of the j^th^ reactant, respectively. A link between reaction reversibility and concentrations of metabolic reactants and products can be drawn (Eqns. S3-S4), and illustrates how metabolite concentrations control reaction reversibility, and, by extension isotope fractionation [46]:

$f_{p,r}= \frac{\Pi_{i}\left[ p_{i} \right]^{m_{i}}}{\Pi_{j}\left[ r_{j} \right]^{n_{j}}} \times e^{\frac{\Delta G_{r}^{o}}{\mathrm{RT}}}$ (S3)

and

$f_{p,r}= \frac{\Phi_{p,r}}{\Phi_{r,p}}$ . (S4)

This model was built to describe sulfur isotope fractionations, but since the sulfur and the oxygen isotope systems share the same metabolic network, we directly adapt the Wing and Halevy (2014) to reflect oxygen isotope fractionations. To maintain consistency in mass balance between the two systems, the step-specific $f$ values we employ in our version of the Wing and Halevy (2014) model are the same as for the sulfur isotope version where fluxes are shared. The thermodynamic driving force is thus the same for both the sulfur and oxygen systems.

**Temperature-dependent equilibrium fractionation factors**

For equilibrium effects, both experimental and theoretical work provide the water - sulfite equilibrium [45]:

${}^{18}{\alpha_{sulfite, water}^{\mathrm{eq}}}=\frac{13.61-0.299 \times pH-0.081 \times(T_{K}-273)}{1000}+1 ,$ (S5)

and the water - sulfate equilibrium [48]:

${}^{18}{\alpha_{sulfate, water}^{\mathrm{eq}}}=\frac{2.72 \times\frac{{10}^{6}}{T_{K}^{2}}-7.71}{1000}+1 .$ (S6)

In considering previous analyses [45, 34], we assume instantaneous equilibrium between sulfite and water, resulting in a $f_{sulfite, water}$ value of 1, which anchors the downstream oxygen isotope composition to that of water.

The oxygen isotopic composition of intracellular phosphate results from a temperature and pH

dependent equilibrium effect with water. We use a definition modified from [32]:

${}^{18}{\alpha_{phosphate, water}^{\mathrm{eq}}}=\frac{1}{{}^{18}{R_{\mathrm{water}}}} \times\left( \frac{\frac{111.4-T}{4.3} + \left( {}^{18}{R_{\mathrm{water}}}-1 \right) \times1000}{1000}+1 \right)$ (S7)

and insert this isotopic offset into the analysis of APS cycling. The isotopic composition of intracellular APS reflects that of phosphate and sulfite, in a 1:3 ratio.

**Model solution**

Here are the reservoir specific solutions that can then be nested for the full solution.

**Intracellular sulfite**

${}^{18}{R_{SO3}}={}^{18}{R_{H2O}} \times\left( {}^{18}{\alpha_{SO3,H2O}^{\mathrm{kin}}} \times\left( 1-f_{SO3,H2O} \right)+{}^{18}{\alpha_{SO3,H2O}^{\mathrm{eq}}} \times f_{SO3,H2O} \right)$ (S8)

Here:

${}^{18}{R_{SO3}}={}^{18}{R_{H2O}} \times{}^{18}{\alpha_{SO3,H2O}^{\mathrm{eq}}}$ (S9)

**Intracellular APS**

${}^{18}{R_{\mathrm{APS}}}={}^{18}{\alpha_{APS red., forward}^{\mathrm{kin}}} \times\left( {}^{18}{R_{H2O}} \times\left( 1-f_{APS,SO3} \right)+ f_{APS,SO3} \times\left( \frac{3}{4} \times{}^{18}{R_{SO3}}+ \frac{1}{4} \times{}^{18}{R_{PO4}} \right) \right)$(S10)

**Intracellular sulfate**

${}^{18}{R_{SO4in}}={}^{18}{\alpha_{APS formation}^{\mathrm{kin}}} \times\left( {}^{18}{R_{H2O}} \times\left( 1-f_{SO4in,APS} \right)+ f_{SO4in,APS} \times\left( F_{\mathrm{APSabio}} \times{}^{18}{\alpha_{enzymatic APS decomposition}^{\mathrm{kin}}} \times{}^{18}{R_{\mathrm{APS}}}+ \frac{9}{10} \times\left( 1- F_{\mathrm{APSabio}} \right) \times\left( \frac{3}{4} \times{}^{18}{R_{\mathrm{APS}}}+ \frac{1}{4} \times{}^{18}{R_{H2O}} \right)+ \frac{1}{10} . \left( 1- F_{\mathrm{APSabio}} \right) \times{}^{18}{R_{\mathrm{APS}}} \right) \right)$ (S11)

**Extracellular sulfate**

${}^{18}{R_{SO4out}}={}^{18}{R_{H2O}} \times{}^{18}{\alpha_{sulfate uptake}^{\mathrm{kin}}}\times\left( 1-f_{SO4out,SO4in} \right)+ \frac{{}^{18}{\alpha_{sulfate uptake}^{\mathrm{kin}}}}{{}^{18}{\alpha_{sulfate export}^{\mathrm{kin}}}} \times f_{SO4out,SO4in} \times{}^{18}{R_{SO4in}}$ (S12)

**Aarhus Bay rate inferences**

We first extract pore water cell-specific sulfate reduction rates using sulfate and sulfide concentrations, and the sulfate δ^18^O reported [50]. The main input into our biochemically informed, thermodynamically driven cellular-scale model is the degree of reaction reversibility ($f$ values) for each step of the network (Eqn. S4). In addition, as described above, reaction reversibility also encapsulates the thermodynamics of the reaction (Eqn. S3). Thus, $f$ depends on (1) environmental (sulfate and sulfide concentrations), and (2) physiological (cell specific rates of sulfate reduction) conditions. Environmental conditions are directly measured in pore waters.

We performed another nonlinear least-squares analysis for each depth-dependent sulfate δ^18^O reported [50], this time using the magnitude of each kinetic isotope effect determined above and solving for cell-specific sulfate reduction rates. For this, we first create a dataset of possible depth-dependent sulfate δ^18^O values for a range of cell-specific sulfate reduction rates, using the pore water sulfate/sulfide concentrations measured at site M1 as additional constraints. We solve for csSRR values for specific pore water sulfate/sulfide concentrations that minimize the residual between observed and predicted sulfate δ^18^O values. To maintain consistency with our earlier conclusions on increased involvement of APS hydrolysis as csSRR decrease, we set F_APS_ equal to 0.

**Unit conversions**

In our model, rates are expressed in fmol H_2_S per cell per day. Diagenetic models, on the other hand, express rates in μmol S per cm^3^ per year. We thus perform the following conversion:

$\frac{\mathrm{fmol}}{day x cell} \to\frac{\mu mol}{year x \mathrm{cm}^{3}}$ (S13)

We turn fmol H_2_S per cell per day into μmol S per cell per year using the following:

$1 fmol= {10}^{-9} \mu mol$ (S14)

$1 day= \frac{1}{365}\mathrm{years}$ (S15)

To convert cell numbers into cm^3^, we turn to using sediment cell abundances. Indeed, performing a direct unit conversion would not be wise, as it would imply sediments composed exclusively of sulfate reducing bacteria, consequently overestimating the sulfate reduction rates. Sediment cell abundances are expressed in cells per cm^3^. We scale sulfate reduction rates in μmol S per cell per year using sedimentary cell abundances that, when applied to our unit conversion to yield rates of sulfate reduction in μmol S per cm^3^ per year that match those inferred by organoclastic sulfate reduction model produced for site M1 [50].

**Intracellular dynamics: Extent of intracellular reoxidation**

In this section, we will quantify the magnitude of the total reoxidation fluxes relative to net sulfide production (that is, cell-specific sulfate reduction rates). In other words, we are asking how much sulfate is reset with respect to oxygen isotopes, and whose signature is effectively recorded in the final extracellular sulfate oxygen isotopic compositions. This value, when applied to global sulfate reduction rate estimates, will enhance our understanding of the environmental information enclosed in porewater sulfate δ^18^O trends in both modern and paleo-environments.

We consider material that has reached the sulfite reservoir, and hence equilibrated with water. For reference throughout this section, the pathway and numbering is:

${\mathrm{SO}_{4}^{2-}}_{\mathrm{ext}} \begin{matrix} J_{1} \\ \rightleftharpoons\\ J_{2} \end{matrix} {\mathrm{SO}_{4}^{2-}}_{\mathrm{int}} \begin{matrix} J_{3} \\ \rightleftharpoons\\ J_{4} \end{matrix}\mathrm{APS}\begin{matrix} J_{5} \\ \rightleftharpoons\\ J_{6} \end{matrix} \mathrm{SO}_{3}^{2-} \begin{matrix} J_{5} \\ \rightleftharpoons\\ J_{6} \end{matrix} H_{2}S ,$ (S16)

and where $f_{x}$values are defined as usual. For those, reaction '$x$' references $J_{1} : J_{2}$ and so on.

The oxidative flux away from sulfite ( $J_{6}$, in mol/time) is taken as the largest possible mass flux that can modify extracellular sulfate (i.e. if all that oxidized sulfite goes quantitatively to sulfate). This flux is then modified by the 'likelihood' that the $J_{6}$ material actually makes it back to sulfate. This draws in the two conversions and three reservoirs that reside upstream of $J_{6}$. We solve for the mass balance on the reservoir being acted upon $\frac{outflux-of-choice}{sum-of-outfluxes}$ . This would take the form:

$F_{SO3}= \frac{J_{6} \times\frac{J_{4}}{J_{4}+ J_{5}} \times\frac{J_{2}}{J_{2}+ J_{3}}}{\mathrm{csSRR}}$ (S17)

The solutions for the oxidative fluxes are rooted in the Wing and Halevy model [46] and thus variable as a function of environmental conditions and metabolic rate. With that, we use the cell-specific sulfate reduction rates determined using the thermodynamically informed model for oxygen isotope effects during MSR and explore the depth-specific values taken by $F_{SO3}$ at site M1 in Aarhus Bay. Results are shown in Figure S8, which depicts the $F_{SO3}$ depth profile (Figure S8.A), and its relationship to cell-specific sulfate reduction rates (Figure S8.B), and sulfate/sulfide concentrations (Figure S8.C).

**2 Legends for SOM tables and figures**

**Figure S1:** Full illustration of the dissimilatory sulfate reduction network used in our thermodynamically informed metabolic model. It emphasizes the location and destination of specific oxygen atoms during forward and reverse reaction, here split for clarity of illustration.

**Figure S2:** Oxygen and sulfur isotope fractionation observed during *Desulfovibrio vulgaris* (red circles) low growth rate and low sulfate chemostat experiments, as well as *Desulfovibrio alaskensis* (blue circles) low sulfate chemostat experiments. The size of the circles corresponds to the sulfate concentrations in the feed media, as shown in the figure's legend.

**Figure S3:** Water normalized oxygen isotopic composition of extracellular sulfate for all chemostat experiments. We also fit a Gaussian curve overlying the data to highlight the nature of the distribution.

**Figure S4:** Residence time of intracellular sulfite (in seconds) for each chemostat experiment as a function of cell-specific sulfate reduction rates (in fmol H_2_S per cell per day).

**Figure S5:** Effect of APS hydrolysis on extracellular sulfate oxygen isotopic compositions.

**Figure S6:** Residence time of intracellular APS (in seconds) for each chemostat experiment as a function of cell-specific sulfate reduction rates (in fmol H_2_S per cell per day).

**Figure S7:** Field of values (focused on the range between +10 and +25 ‰) of sulfate δ^18^O for a range of extracellular sulfate and sulfide concentrations. The column on the left shows results for cell-specific sulfate reduction rates at 1 fmol H2S per cell per day, the column on the right, results are shown for 10 fmol H_2_S per cell per day. Here, we show solutions when using the median value of step-specific isotope effects, as well as the upper and lower bounds defined by their respective 68 and 95 % confidence intervals. Temperature, and the oxygen isotopic composition of ambient water and intracellular phosphate are adjusted to reflect pore water conditions. The back-reaction of APS to intracellular sulfate is set to be dominated by abiotic APS hydrolysis, as per our inferences from its relation to cell-specific sulfate reduction rates from figure 2.

**Figure S8:** Measured [50] and predicted oxygen isotopic compositions of pore water sulfates in site M1 in Aarhus Bay. A line with a slope of 1 is shown as reference (gray line). Median, and 68 % confidence intervals are superimposed. For these, the back-reaction of APS to intracellular sulfate is set to be dominated by abiotic APS hydrolysis, as per our inferences from its relation to csSRR from figure 2.

**Figure S9:** Extent of intracellular reoxidation ($F_{SO3}$) at site M1 in Aarhus Bay. **A:** Depth-specific of $F_{SO3}$ values. The red line indicates values determined using the median values of cell-specific sulfate reduction rates determined using the thermodynamically informed model for oxygen isotope effects during MSR applied to site M1, and the dark gray and the light areas represent the 68 and 95 % confidence interval on said values, respectively. **B:** $F_{SO3}$ values, as determined using the median values of cell- specific sulfate reduction rates, as a function of the corresponding cell-specific sulfate reduction rates. **C:** $F_{SO3}$ values, as determined using the median values of cell-specific sulfate reduction rates, as a function of the corresponding depth-specific sulfate to sulfide ratios.

**Figure S10:** Depth profile of major sulfur isotope fractionation factor for the Aarhus Bay profile at site M1. Observed (dark green circles) and predicted values based on depth-specific cell-specific sulfate reduction rates and sulfate/sulfide concentrations are shown. The red line shows values calculated using median values of sulfate reduction rates, and gray areas correspond to 68 and 95 % confidence intervals.

**Table S1:** Oxygen isotopic compositions of extracellular sulfate for each of the pure culture chemostat experiments. Strain, ambient sulfate concentrations (expressed in mM), and cell-specific sulfate reduction rates are shown.

**Table S2:** Results of the two-tailed hypothesis test for the effect of sulfate concentration, cell-specific sulfate reduction rate, and strain of sulfate reducing bacterium on the MSR-induced sulfate oxygen isotopic composition.
