# Supplementary figures and images for: Oxygen isotope effects during microbial sulfate reduction: applications to sediment cell abundances
Source: ISME J. 2020 Mar 9;14(6):1508–19. doi: 10.1038/s41396-020-0618-2 (PMC7242377; doi:10.1038/s41396-020-0618-2)

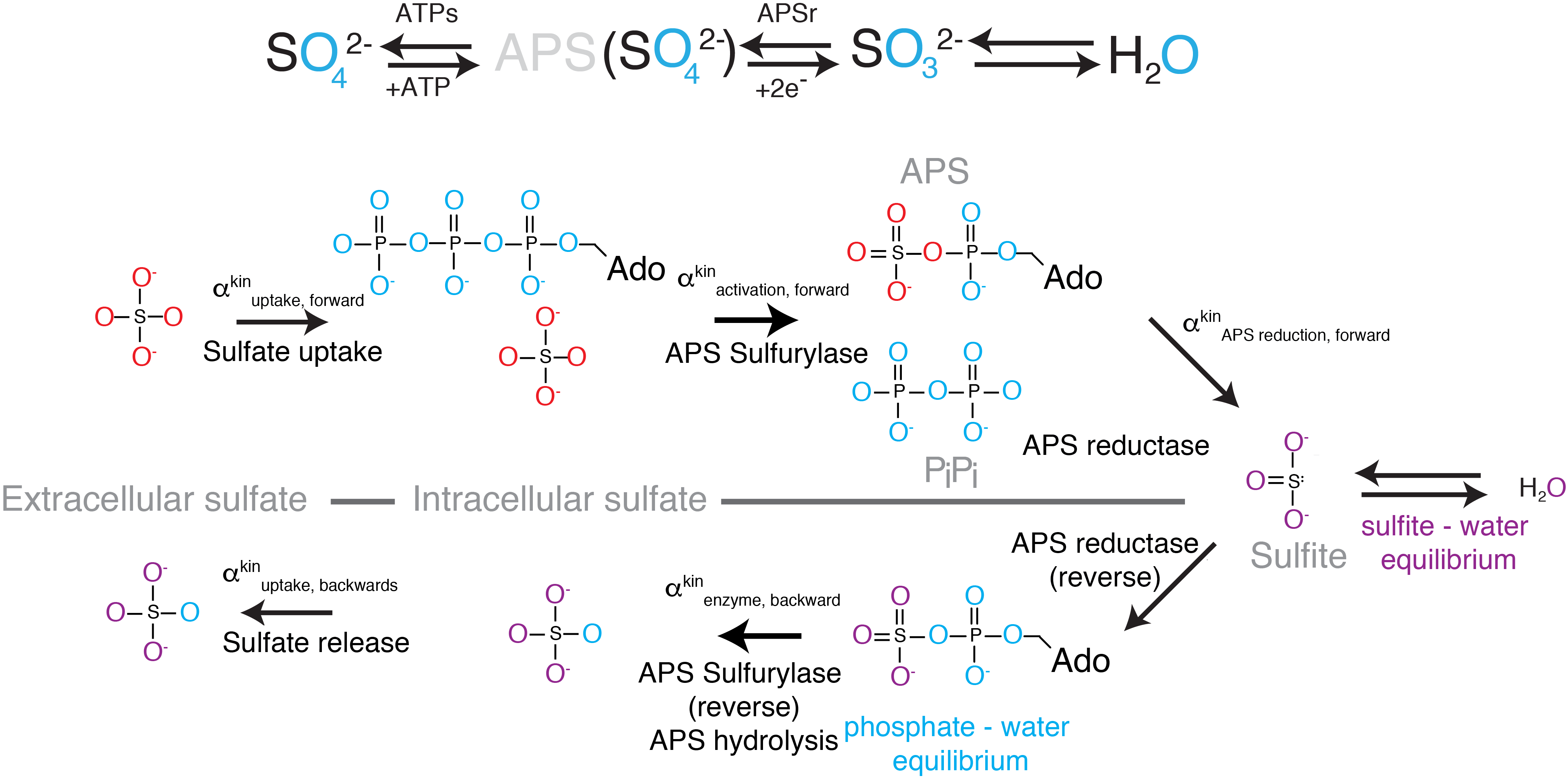

Supplement: Supplementary file 2 — Supplementary Figure 1 [file 41396_2020_618_MOESM2_ESM.png]

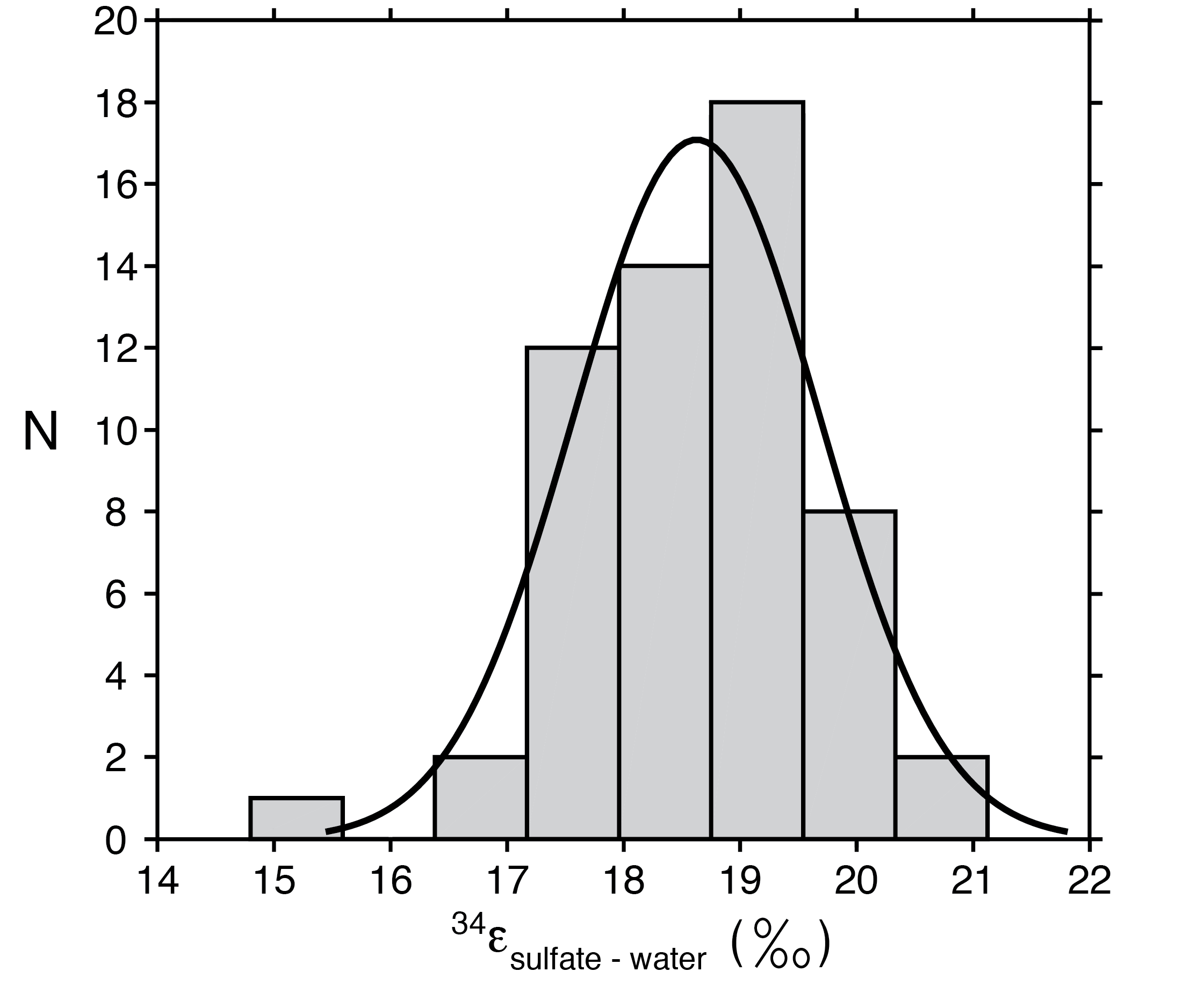

Supplement: Supplementary file 4 — Supplementary Figure 3 [file 41396_2020_618_MOESM4_ESM.png]

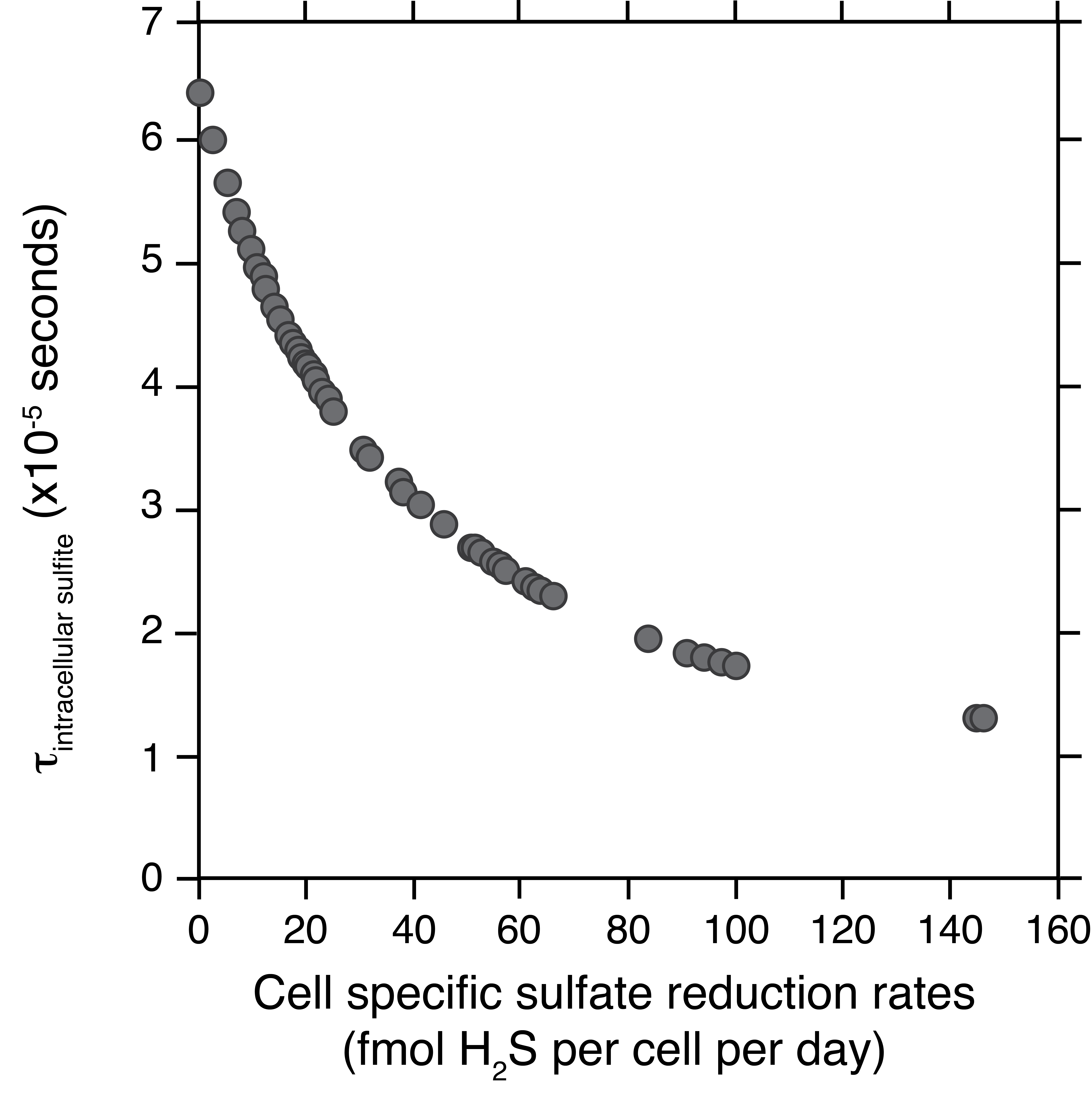

Supplement: Supplementary file 5 — Supplementary Figure 4 [file 41396_2020_618_MOESM5_ESM.png]

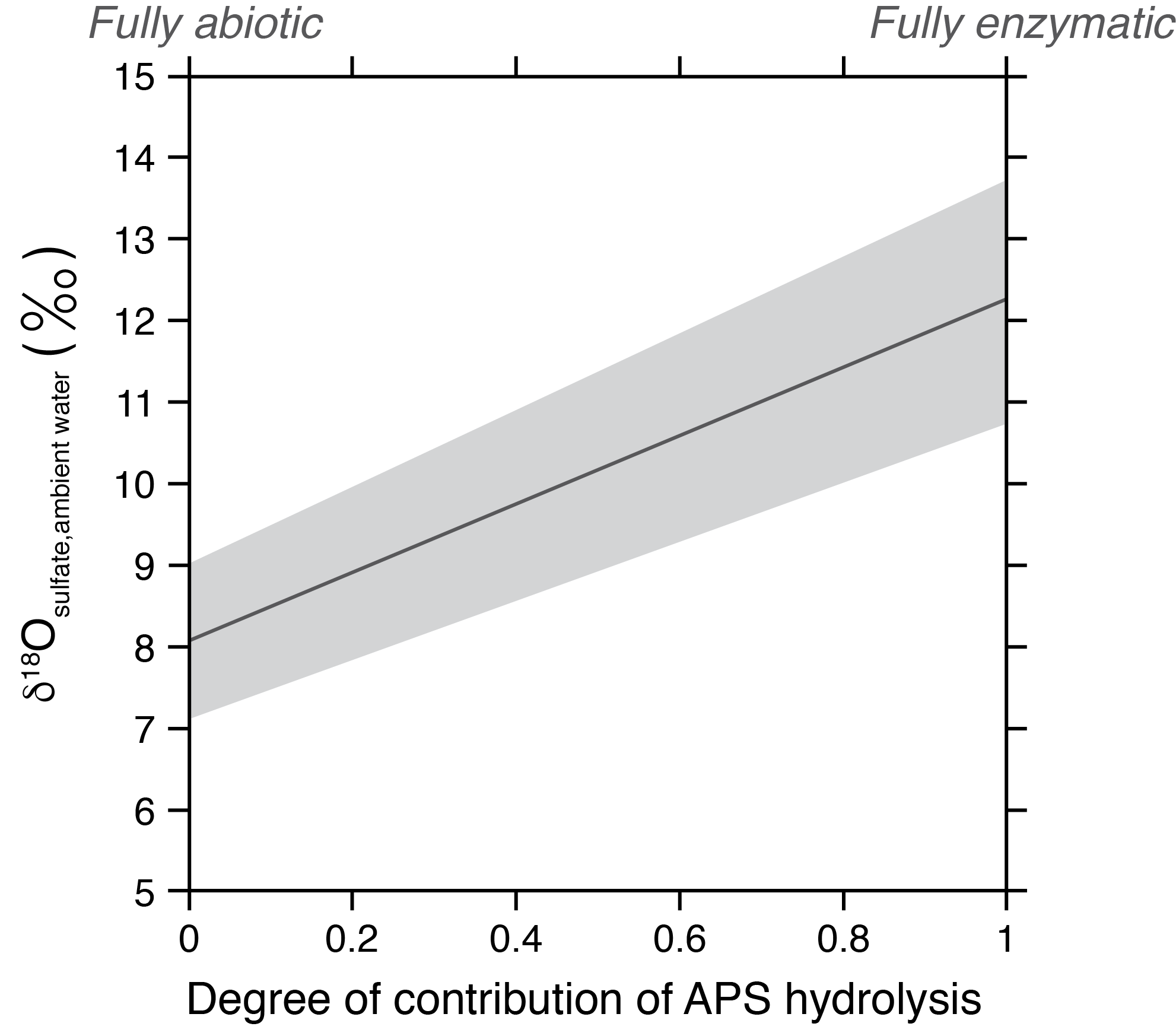

Supplement: Supplementary file 6 — Supplementary Figure 5 [file 41396_2020_618_MOESM6_ESM.png]

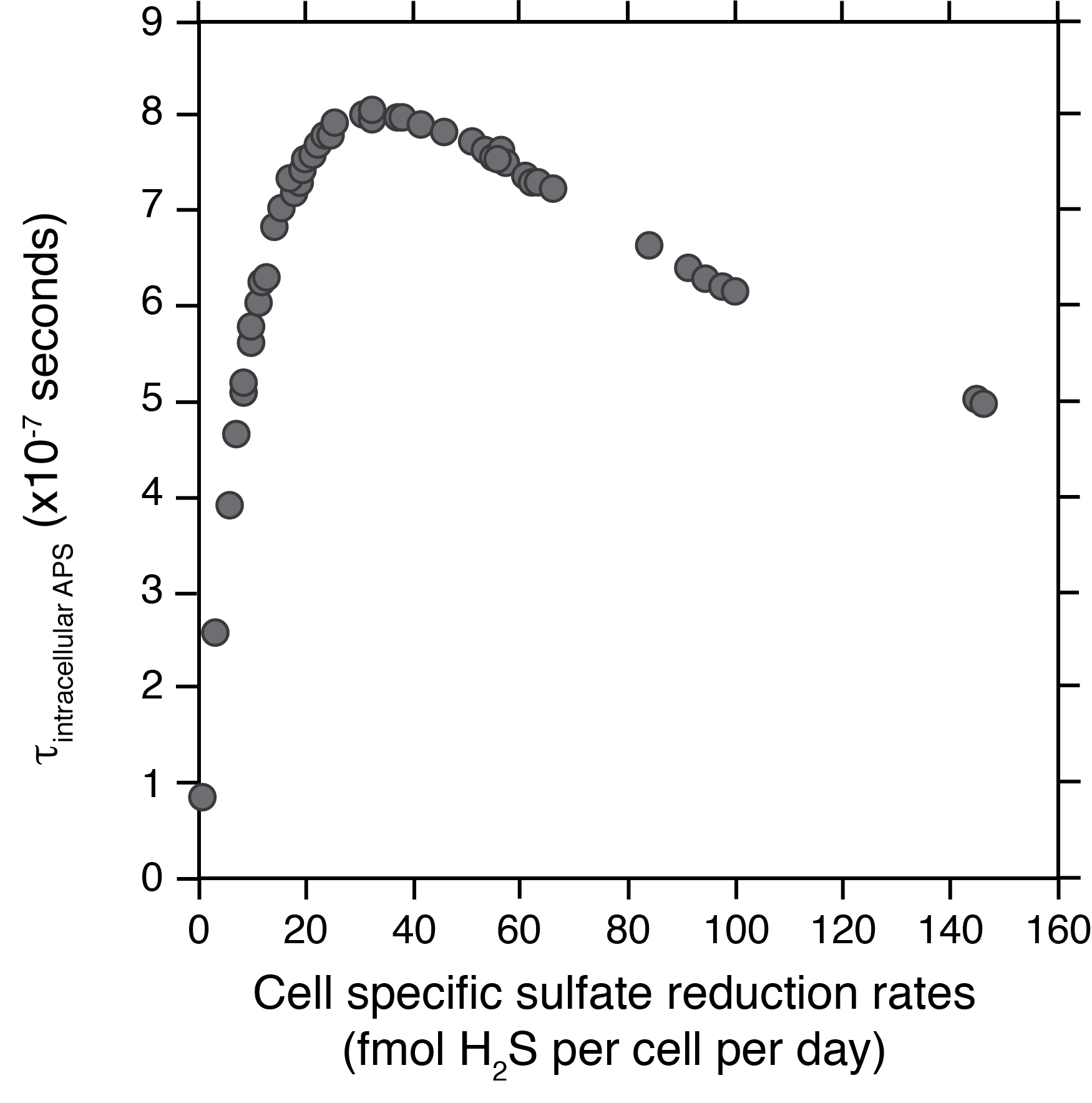

Supplement: Supplementary file 7 — Supplementary Figure 6 [file 41396_2020_618_MOESM7_ESM.png]

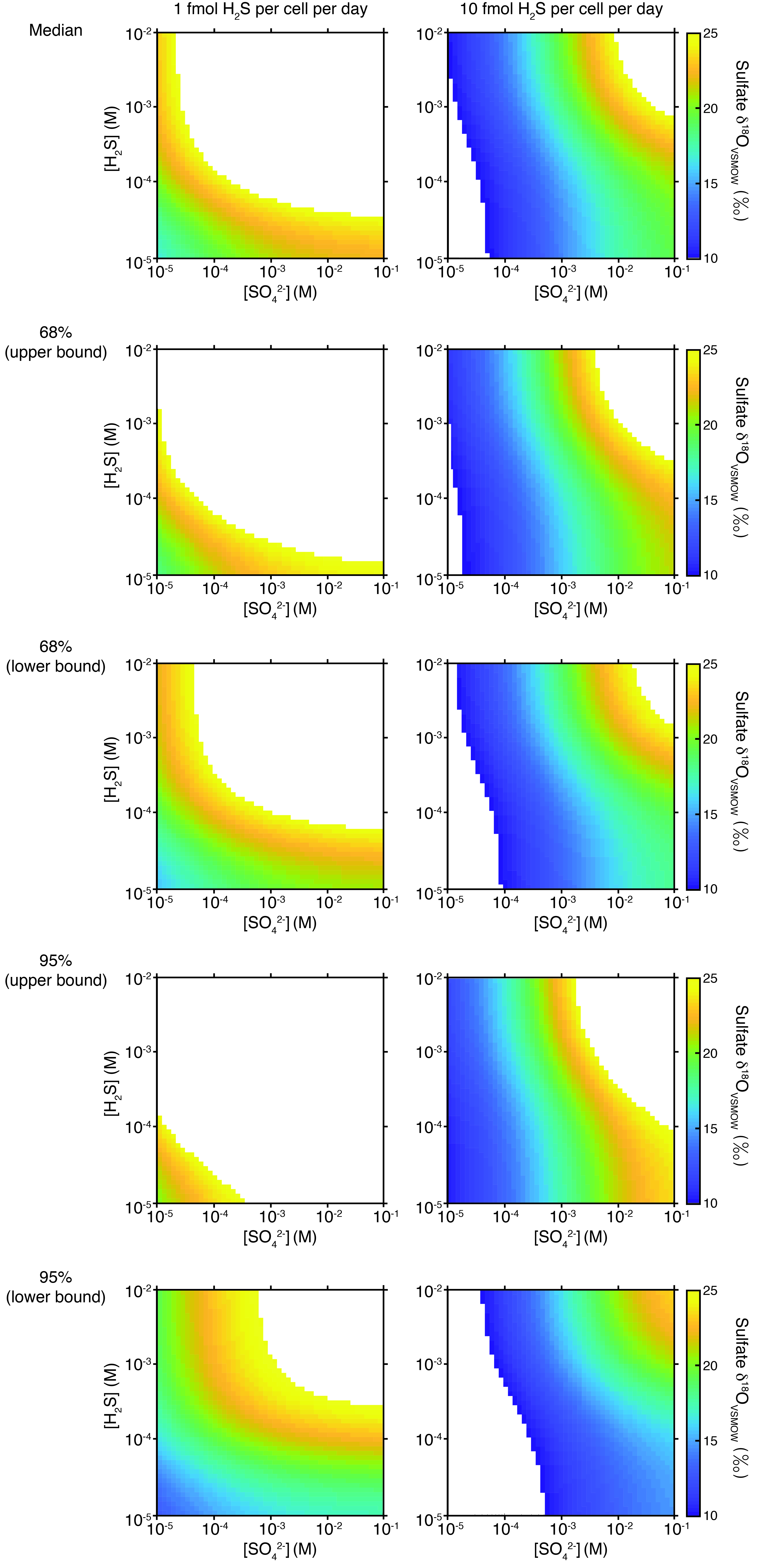

Supplement: Supplementary file 8 — Supplementary Figure 7 [file 41396_2020_618_MOESM8_ESM.jpg]

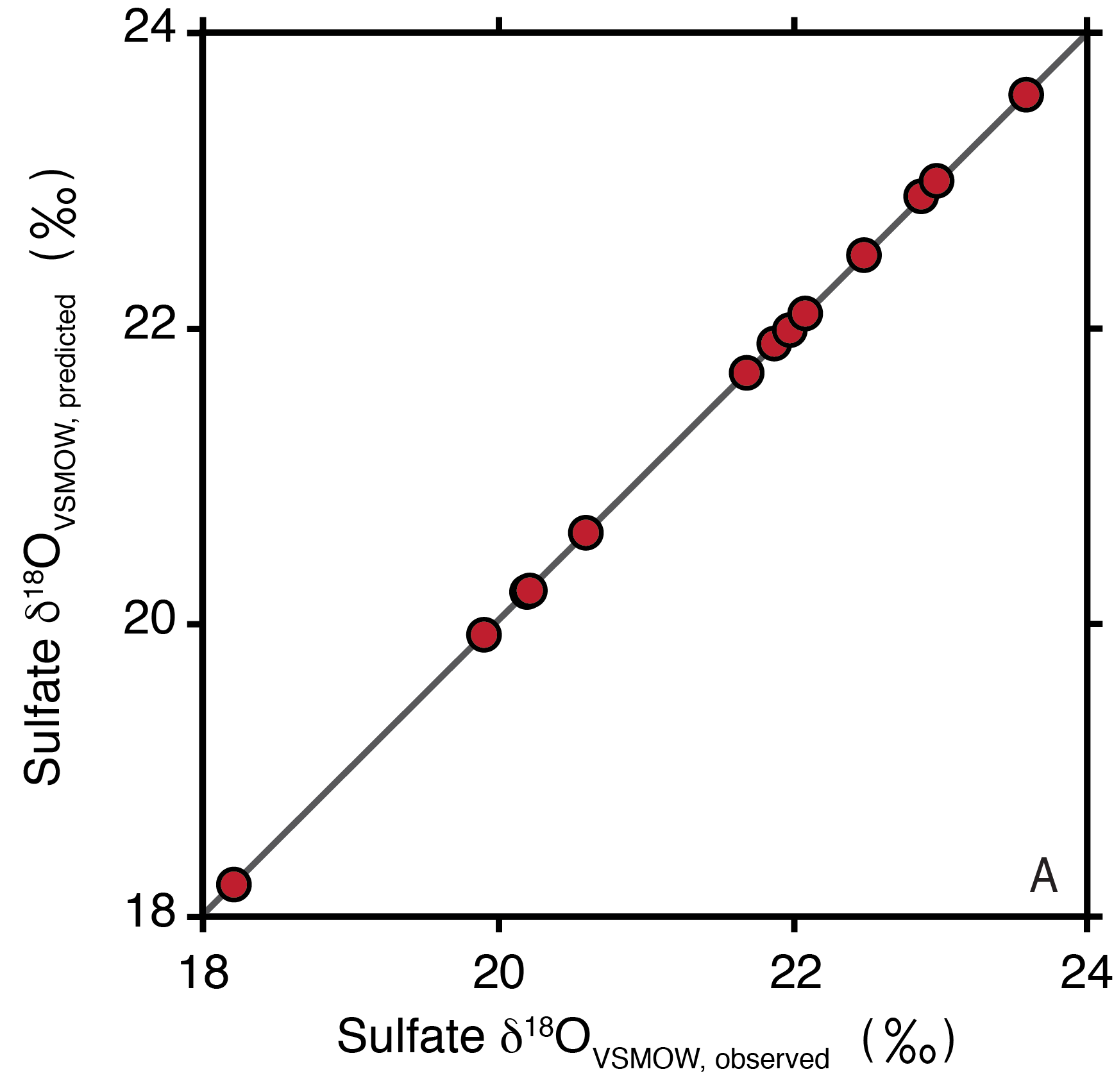

Supplement: Supplementary file 9 — Supplementary Figure 8 [file 41396_2020_618_MOESM9_ESM.png]

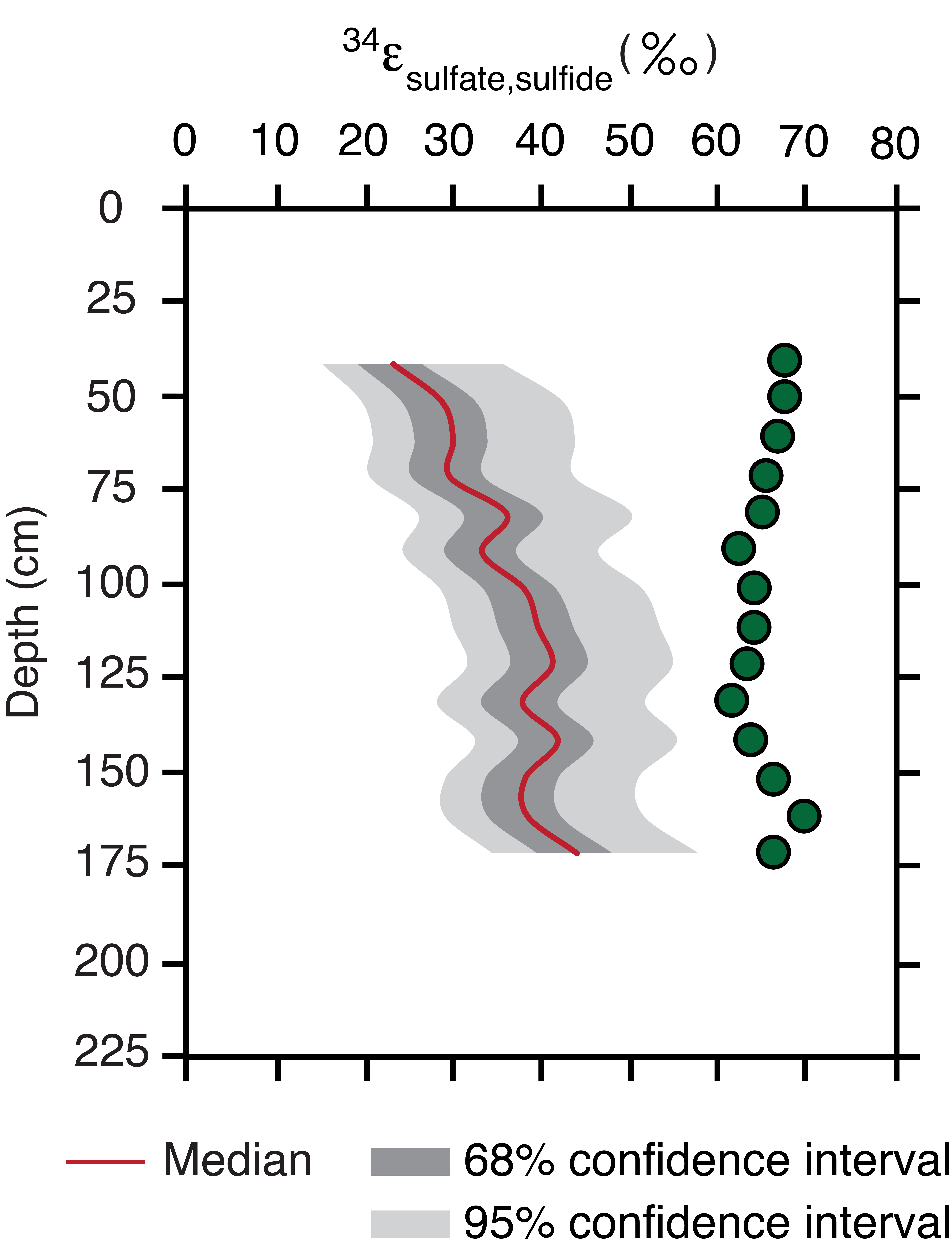

Supplement: Supplementary file 11 — Supplementary Figure 10 [file 41396_2020_618_MOESM11_ESM.png]
