# Supplementary material for: Oxygen isotope effects during microbial sulfate reduction: applications to sediment cell abundances
Source: ISME J. 2020 Mar 9;14(6):1508–19. doi: 10.1038/s41396-020-0618-2 (PMC7242377; doi:10.1038/s41396-020-0618-2)
Supplement: Supplementary file 12 — Supplementary Table 1 [file 41396_2020_618_MOESM12_ESM.docx]

| **Strain** | **[SO4]out**  **mM** | **csSRR**  **fmol H_2_S per cell per day** | **^18^ε_sulfate,ambientwater_**  **per mil** |
| --- | --- | --- | --- |
| *D. vulgaris*  str. Hildenborough | 1 | 17.482 | 18.3 |
|  |  | 17.813 | 16.8 |
|  | 2 | 10.183 | 17.2 |
|  |  | 11.554 | 17.3 |
|  | 5 | 18.034 | 18.2 |
|  |  | 18.814 | 17.6 |
|  |  | 19.719 | 20.6 |
|  |  | 20.911 | 17.9 |
|  |  | 21.649 | 18.4 |
|  |  | 22.187 | 16.8 |
|  |  | 23.222 | 17.2 |
|  |  | 24.424 | 17.9 |
|  | 28 | 0.924 | 19.2 |
|  |  | 3.349 | 19.3 |
|  |  | 3.368 | 19.6 |
|  |  | 5.832 | 19.3 |
|  |  | 13.032 | 19.4 |
|  |  | 32.561 | 19.4 |
|  |  | 37.325 | 18.9 |
|  |  | 38.371 | 18.8 |
|  |  | 41.788 | 19.2 |
|  |  | 42.063 | 18.7 |
|  |  | 46.149 | 18.8 |
|  |  | 51.34 | 18.7 |
|  |  | 51.419 | 19.6 |
|  |  | 53.435 | 18.9 |
|  |  | 55.536 | 18.6 |
|  |  | 56.561 | 19 |
|  |  | 57.735 | 18.9 |
|  |  | 61.267 | 19 |
|  |  | 62.929 | 19.2 |
|  |  | 63.497 | 19.9 |
|  |  | 66.582 | 19.6 |
|  |  | 84.39 | 19 |
|  |  | 91.454 | 19.1 |
|  |  | 91.524 | 19.7 |
|  |  | 94.59 | 20 |
|  |  | 97.937 | 19.9 |
|  |  | 100.705 | 20.3 |
|  |  | 145.322 | 19.4 |
|  |  | 146.702 | 18.7 |
| *D. alaskensis*  str. G-20 | 0.5 | 10.111 | 18.8 |
|  | 1 | 8.715 | 18 |
|  |  | 12.162 | 21.1 |
|  |  | 14.664 | 14.8 |
|  |  | 15.928 | 18.4 |
|  | 2 | 17.498 | 18.2 |
|  |  | 20.247 | 18 |
|  |  | 22.447 | 17.8 |
|  |  | 24.117 | 17.8 |
|  |  | 25.647 | 18.6 |
|  | 5 | 7.507 | 17.8 |
|  |  | 8.644 | 17.8 |
|  |  | 25.295 | 18.4 |
|  |  | 31.1 | 17.9 |
|  |  | 32.783 | 18.2 |
|  |  | 56.674 | 17.2 |
