# Supplementary material for: Oxygen isotope effects during microbial sulfate reduction: applications to sediment cell abundances
Source: ISME J. 2020 Mar 9;14(6):1508–19. doi: 10.1038/s41396-020-0618-2 (PMC7242377; doi:10.1038/s41396-020-0618-2)
Supplement: Supplementary file 13 — Supplementary Table 2 [file 41396_2020_618_MOESM13_ESM.docx]

| Test | H_0_ | P-value |
| --- | --- | --- |
| Effect of csSRR on ^18^O_sulfate_ as a function of rate is zero | The slope of ^18^O_sulfate_  (average ^18^O_sulfate_ /csSRR) | 0.99 |
| Effect of [SO4] on ^18^O_sulfate_ (difference between average ^18^O_sulfate_ between experiments) | Between 28 mM and 1 mM sulfate | 0.94 |
|  | Between 28 mM and 2 mM sulfate | 0.93 |
|  | Between 28 mM and 5 mM sulfate | 0.94 |
|  | Between 28 mM and 0.5 mM sulfate | 0.98 |
|  | Between 1 mM and 2 mM sulfate | 0.99 |
|  | Between 1 mM and 5 mM sulfate | 0.99 |
|  | Between 1 mM and 0.5 mM sulfate | 0.96 |
|  | Between 2 mM and 5 mM sulfate | 0.99 |
|  | Between 2 mM and 0.5 mM sulfate | 0.95 |
|  | Between 5 mM and 0.5 mM sulfate | 0.96 |
| Effect of bacterial strain on ^18^O_sulfate_ | The difference between the average ^18^O_sulfate produced_ by *D. vulgaris* str. Hildenborough and *D. alaskensis* str. G-20 is zero | 0.96 |
